# Impact of Land Urbanization on Carbon Emissions in Urban Agglomerations of the Middle Reaches of the Yangtze River

**DOI:** 10.3390/ijerph18041403

**Published:** 2021-02-03

**Authors:** Di Zhang, Zhanqi Wang, Shicheng Li, Hongwei Zhang

**Affiliations:** Department of Land Resource Management, School of Public Administration, China University of Geosciences (Wuhan), Wuhan 430074, China; dzhang9240@163.com (D.Z.); lisc@cug.edu.cn (S.L.); zhangfocus@cug.edu.cn (H.Z.)

**Keywords:** carbon emission, land urbanization, decomposition analysis, the middle reaches of the Yangtze River, MYR-UA

## Abstract

The urban agglomerations in the middle reaches of the Yangtze River (MYR-UA) are facing a severe challenge in reducing carbon emissions while maintaining stable economic growth and prioritizing ecological protection. The energy consumption related to land urbanization makes an important contribution to the increase in carbon emissions. In this study, an IPAT/Kaya identity model is used to understand how land urbanization affected carbon emissions in Wuhan, Changsha, and Nanchang, the three major cities in the middle reaches of the Yangtze River, from 2000 to 2017. Following the core idea of the Kaya identity model, sources of carbon emissions are decomposed into eight factors: urban expansion, economic level, industrialization, population structure, land use, population density, energy intensity, and carbon emission intensity. Furthermore, using the Logarithmic Mean Divisia Index (LMDI), we analyze how the different time periods and time series driving forces, especially land urbanization, affect regional carbon emissions. The results indicate that the total area of construction land and the total carbon emissions increased from 2000 to 2017, whereas the growth in carbon emissions decreased later in the period. Energy intensity is the biggest factor in restraining carbon emissions, followed by population density. Urban expansion is more significant than economic growth in promoting carbon emissions, especially in Nanchang. In contrast, the carbon emission intensity has little influence on carbon emissions. Changes in population structure, industrial level, and land use vary regionally and temporally over the different time period.

## 1. Introduction

### 1.1. Background

Countries around the world are at different stages of development in socioeconomics, and rapid urbanization and human interference have contributed to various environmental challenges [1]. According to statistical data from the World Bank (2016) [2], China is rapidly urbanizing, with the proportion of people living in urban areas increasing from 26% in 1991 to 59% in 2018. Meanwhile, the system of land finance leads to land-centered urbanization rather than population-centered urbanization [3]. China’s urbanization has been characterized by a land-centered development pattern centered on the commercialization and expansion of urban land, especially in the provincial capitals [4,5,6]. Land urbanization is the process of land pattern change, which mainly entails the increase of construction land in that the total land area remains unchanged in a region [7]. As a result, intense urban expansion has been a significant symptom of the rapid land urbanization [8]. Many ecological lands have become occupied accompanying this large-scale [9], rapid urban expansion, which has also reduced the utilization rate of land resources. The demand for energy consumption has also increased significantly, driven by demography and economy [10]. Despite the fact that urban development can increase residents’ income and improve people’s living standards, it must be recognized that such growth can also lead to an increase in energy consumption. Long-term maintenance of land-centered urbanization is not only inefficient but also places great pressure on resources and the environment, which impedes the sustainable development of the region [11,12]. In particular, the increase in greenhouse gas emissions, mainly composed of carbon emissions, contributes to global warming, which can imperil the environment and threaten economic activities.

According to the Intergovernmental Panel on Climate Change (IPCC), the rising carbon dioxide concentrations in the atmosphere will increase the average global temperature and cause negative consequences for the environment, as well as irreversible damage in the long term [13]. In 2013, China’s carbon emissions reached 25% of the global total, 1.5 times that of the United States [14,15]. Under the premise of maintaining economic development, China is faced with arduous ecological and environmental challenges. To address the problem of global warming, the United States, the European Union, Japan, and other countries have established a shared global vision of a long-term carbon reduction strategy. To keep the carbon dioxide concentration in the atmosphere below 450 ppm by 2050 and to keep the global temperature rise at the end of this century at less than 2 °C [16,17], China plans to reduce its emissions intensity by 40–45% in 2020 compared with 2005 levels [18]. Reaching this target in emissions reduction requires not only implementing the strategic plan on the macro national level but also sharing the responsibility among the regions on the micro-level. Cities are the centers of livelihood and production and, with their economic development and high creativity, are being recognized as important regional units for the implementation of carbon emission mitigation policies.

The urban agglomeration in the middle reaches of the Yangtze River (MYR-UA) is the key area in the strategy of the central region. The mode of economic development, industrial structure, and residential living standards has changed under the influence of the current urbanization and industrialization. These changes may directly or indirectly lead to increases in urban carbon emissions, which would further worsen the ecological impact. In 2016 in particular, the Chinese government put forward the priority requirement of environmental protection in the Yangtze River economic belt [19], so the issue of carbon emissions has attracted increased attention. To effectively control and mitigate carbon emissions, we need to analyze the factors that affect such emissions. Thus, we must understand the carbon emission characteristics of typical cities undergoing rapid urbanization and further analyze the factors affecting carbon emissions to arrive at significant policies for reducing emissions.

### 1.2. Literature Review

Previous studies have discussed issues related to carbon emission trends and characteristics, as well as the harm done to the economy and to the human environment. In recent years, increasing attention has concentrated on analyzing factors that affect carbon emissions. Among these determinants, urbanization results in higher energy consumption and carbon emissions [20]. With the process of urbanization, some studies have paid significant attention to the relationship between urbanization and carbon emissions, and established models of the relationship between them [21]. Liu et al. found that urbanization increased carbon emissions in China and its eastern, central, and western regions, and observed a unidirectional causation running from land urbanization to carbon emissions in the eastern and central regions in the short term [22]. Wang et al. investigated the multiple effects of urbanization on carbon emissions in the Pearl River Delta of China’s economically developed regions from 1990 to 2013, from the four different aspects of economy, population, land, and society. The results show that land urbanization positively affects emissions due to transformation from non-built to built-up area [8], which is consistent with the finding of Zhou et al. [23]. In addition to these, other studies discuss the relationship between urbanization variables and carbon emissions from the perspective of population. Zhang et al. showed that an inverted U-shaped relationship between urbanization and carbon emissions exists by using panel data of 141 countries from 1961 to 2011 [24]. Decomposing the change in carbon emissions into some influencing factors caused by urbanization, Li et al. found that the contribution of total population is not absolute nor is it obvious due to its small change during the period 1990–2012 [25]. Besides, other research has investigated the profound impacts of urbanization on CO_2_ emissions while considering the integrated evolution of urbanization. Results have documented that improvements in the urbanization quality have contributed to reducing CO_2_ emissions [26]. Many studies, such as Zhang et al. [24], Ding et al. [27], and Gong et al. [28], describe urbanization in terms of rapid increase in the proportion of urban population. The different comprehensive effects from urbanization’s various aspects may have different impacts on the dynamic changes of carbon emissions [29]. Therefore, analyzing the impact of urbanization on carbon emissions requires a more thorough examination. One must also pay attention to the process of land urbanization, because the transformation of land into urban construction land may have an important impact on China’s urbanization [7]. Most studies analyze the factors that affect carbon emissions at the national level [30], which makes it easier to compare countries. Recently, some studies have focused on the sub-national level to refine emission reduction plans [31,32,33,34], and have found that both the influencing directions and magnitudes of economic development and industrial structure on carbon emissions varied widely by region. According to Feng et al., the developed regions showed larger growth rates of carbon emissions owing to their greater changes in urbanization and associated lifestyle despite utilizing advanced technologies [35]. These studies have proposed recommendations to implement regional climate policies in line with regional features and development realities. Given the wide development of urban agglomerations, research into urban carbon emissions has received more attention [36,37,38]. Economic development, population growth, industrial structure, and urban population scale were found to positively drive the energy consumption and promote carbon emissions at the city level [39,40]. Compared with other cities in the urban agglomerations, the proportion of carbon dioxide emissions in provincial capital cities has increased [41]. Literature review reveals that the majority of studies that measure effects of urbanization on carbon emissions consider the global and national macro-scales or the provincial scale, and fewer studies look at urban agglomerations [42].

Index decomposition analysis (IDA), which uses index number theory in decomposition, is a commonly used method for scientific evaluation and quantitative analysis of factors affecting carbon emissions [43,44,45]. Based on the available data at any aggregation level, this method analyzes the direct and indirect effects of various factors on carbon emissions over a given period or temporal time. Analysis of these period and temporal decompositions helps us understand what causes carbon emissions to change over time. The Logarithmic Mean Divisia Index (LMDI), one type of IDA which does not produce a residual term, is more suitable for temporal analysis and has been widely used in the decomposition analysis of carbon emissions [46]. Research that applied the LMDI to decompose CO_2_ emissions focused not only on energy-related and industrial sectors but also on sectors such as transportation, land use, and agriculture [28,31,47,48,49,50,51].

The characteristics of past studies can be summarized as follows: first, the current research focuses mainly on analyzing the factors that affect carbon emissions from a national perspective, or they focus on a province or a few provincial capitals. Fewer studies discuss the regional differences in carbon emissions caused by the many factors that influence MYR-UA. Second, most studies have concentrated on population, industrial, and economic factors that influence carbon emissions, whereas fewer have considered how urban expansion affects carbon emissions.

Thus, considering differences in urbanization may lead to regional differences in carbon emissions, therefore emission reduction in key regions can provide some insights for other regions. Taking as examples Wuhan, Changsha, and Nanchang, the three provincial capitals of the MYR-UA, we analyze, from the perspective of land urbanization, the regional differences in carbon emission characteristics and the factors that cause them for the period 2000–2017.

## 2. Study Area and Methodology

### 2.1. Study Area and Data

**Figure 1:**
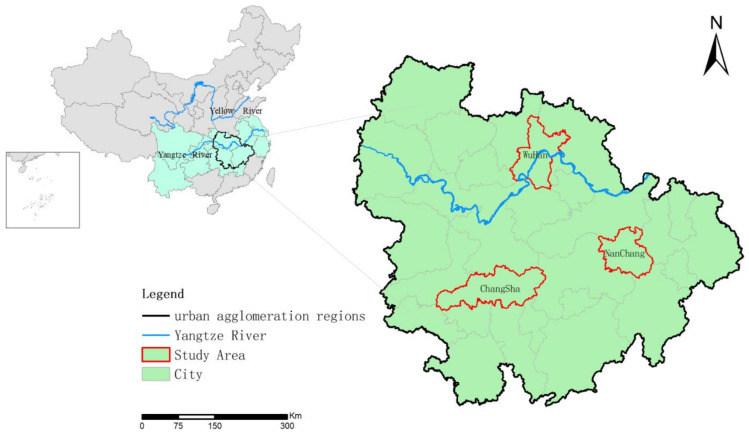
Study area and internal locations.

The MYR-UA is a large-scale urban agglomeration composed of 31 cities. It includes the Wuhan Metropolitan Area, the Changsha–Zhuzhou–Xiangtan urban agglomeration, the Poyang Lake Ecological Economic Zone, and its surrounding cities with Wuhan, Changsha, and Nanchang as the centers (Figure 1). MYR-UA covers an area of 317,000 km^2^, accounting for 3.3% of the total land area of China. It is also a region of agglomerated population and has an advanced economy. In 2014, the GDP and the total population at the end of the year reached 6 trillion yuan and 121 million people, accounting for 8.8% and 8.8% of the national totals, respectively. Moreover, the degree of urbanization of the three urban agglomerations with Wuhan, Changsha, and Nanchang as centers is relatively high, and the urbanization rate of the permanent population exceeded 55% in 2014 [52]. Additionally, the construction land within the urban agglomerations has gradually expanded over the period of this study.

Generally, the energy consumption of different sectors is taken at the emission source to calculate the regional carbon emissions in a study area. The industrial sector accounts for more than 70% of total carbon emissions [53], so we mainly considered energy consumption due to industrial activities on constructed land. The following data sources were used: (1) Energy consumption data of the study area for 2000–2018 from the Statistical Yearbooks of cities, including the Wuhan Statistical Yearbook (Wuhan Statistics Bureau), the Changsha Statistical Yearbook (Changsha Statistics Bureau), and the Nanchang Statistical Yearbook (Nanchang Statistics Bureau) [54,55,56]. Note that electric power consumption and thermal power consumption were not considered here to avoid double accounting. (2) Data for calculations were taken from the 2006 IPCC [57], including the carbon oxidation coefficient, carbon emission coefficient, and the low calorific value. In addition, the consumption of all types of fossil energy was converted into the standard quantity measured by standard coal. The standard coal conversion coefficient comes from the China Energy Statistical Yearbook [58] (it is basically constant, so we used the data from 2017 in this paper), which was compiled by the National Bureau of Statistics of China.

The socio-economic data include gross domestic production (GDP), industrial GDP, a permanent population, and an urban population, which are all available in the regional Statistical Yearbooks [54,55,56]. Built-up areas and urban constructed land areas for the period 2000–2017 were obtained from the China Urban Construction Statistical Yearbook [59]. However, the classification standard of construction land in the statistical yearbook was adjusted in 2011; from nine categories to eight categories. Particularly, construction land includes residential areas, land for administration and public services, commercial and business facilities, industrial and manufacturing, logistics and warehousing, roads and transportation facilities, public utilities, and green space and square (Classification of Urban Land and Planning and Construction Land Standard (GB50137-2011)). The categories were not merged in this study since the sub-classes involved in the classification adjustment are unreachable. To accurately reflect changing economic activity, the nominal GDP of each city from 2000 to 2017 was converted by using the price index deflation method into the fixed price GDP time series with the base year 2000.

### 2.2. Calculation of Carbon Emissions

Carbon emissions from energy consumption account for 90–95% of China’s total carbon emissions, mainly from the daily energy consumption of construction land, including industrial production and transportation [60]. Therefore, the energy consumption of the region was used to estimate the carbon emissions. We used the following formula from the IPCC Guidelines for National Greenhouse Gas Inventories [57]:

(1) $$C^{t}=\sum C_{i}^{t}=\sum E_{i}^{t}{\;\times \;\mathrm{cof}}_{i}^{t}{\;\times \;\mathrm{LCV}}_{i}{\;\times \;O}_{i}$$

where i is the energy type consumed by the various fuels, t is the time of year; $E_{i}^{t}$ is the total energy consumption of various fuels during year t; ${\mathrm{cof}}_{i}^{t}$, ${\mathrm{LCV}}_{i}$, and $O_{i}$ are the carbon emissions coefficient, the lower calorific value, and the oxidation rate for fuel type *i*, respectively; and $C_{i}^{t}$ and $C^{t}$ are the type of energy consumption for carbon emissions and the total energy-related carbon emissions in year *t*.

### 2.3. Decomposition Analysis of Carbon Emissions

#### 2.3.1. Analysis of Driving Factors

To analyze the relevant factors, we used the I=P×A×T formulation (IPAT model), which has been widely discussed for the analysis of carbon emissions. The IPAT model was originally proposed by Ehrlich and Holdren [61] to explain the impact of population and human activity on the environment and its dynamic characteristics in a simple and feasible quantitative method. The basic idea of IPAT model is that the total environmental impacts (I) are a multiplicative function of population size (P), affluence (A, equated as per capita GDP or per capita consumption), and the level of environmental damage caused by technology (T, equated as per unit GDP consumption or production) [62]. In the preceding decades, many studies extended the original IPAT identity from different perspectives and incorporated more factors [63]. The Kaya identity ($CO_{2}=P\times \mathrm{GDP}/P\times E/\mathrm{GDP}\times CO_{2}/E$) [64] is the basis for the Greenhouse Gas emissions calculations that is known as a reformulation of IPAT and was used in this study.

We focused herein on how related factors affect carbon emissions from the point of view of urbanization, especially land urbanization. Consequently, we present a model for a period and for a temporal decomposition of CO_2_ emissions. CO_2_ emissions can be expressed in terms of eight variables as follows:

(2) $${\mathrm{CO}}_{2}{=S}_{B}\times \frac{\mathrm{GDP}}{S_{C}}\times \frac{{\mathrm{GDP}}_{\mathrm{in}}}{\mathrm{GDP}}\times \frac{P_{\mathrm{ur}}}{P}\times \frac{S_{C}}{P_{\mathrm{ur}}}\times \frac{P}{S_{B}}\times \frac{E}{{\mathrm{GDP}}_{\mathrm{in}}}\times \frac{{\mathrm{CO}}_{2}}{E}$$

(3) C=A × G × In × Ps × S × De × EI × CI

where CO_2_ denotes CO_2_ emissions; S_b_ is the area of urban built-up (the built-up area is the range within which the actual developed land of the city exists. Source from China Energy Statistical Yearbook); S_c_ is the area of urban construction land; (the urban construction land refers to area of city except water area and land for other purposes. Source from China Energy Statistical Yearbook. Note that the urban construction land includes expropriated land that has not been used, it may be larger than the built-up area); GDP is the gross domestic product; GDP_in_ is the industrial added value; P is the total population; P_ur_ is the urban population; and E is energy consumption. Equation (2) is balanced on both sides, and the variables on the right-hand side are the main driving forces for CO_2_ emissions, namely, urban expansion (S_b_), economic level (GDP/S_c_), industrialization level (GDP_in_/*GDP*), population structure (P_ur_/P), land-use level (S_c_/P_ur_), population density (P/S_b_), energy intensity (E/GDP_in_), and CO_2_ emission intensity (CO_2_/E). Table 1 lists the definitions of all variables.

The effects of energy intensity, carbon emission intensity, and economic level on CO_2_ emissions have been demonstrated in numerous previous studies [65]. Some report that energy intensity reflects the national technological level to some extent; consequently, the energy intensity has been used to measure technological level, and it mainly measures the comprehensive energy efficiency of a region. A higher energy intensity correlates with a lower regional energy efficiency. The CO_2_ emissions intensity reflects the regional emission efficiency, which is also an important factor affecting CO_2_ emissions. Generally, the economic level is measured by GDP per capita. The GDP per area reflects the density of economic output, which can better reflect the development and economic concentration of a region than GDP per capita [66]. Therefore, considering the construction of a model identity, we measured the level of regional economic development via the ratio of regional GDP to urban construction land. In the process of urbanization, industrial structure and population change cannot be ignored because they significantly impact CO_2_ emissions.

As mentioned above, China’s urbanization in recent years is characterized by population urbanization being slower than the expansion of urban land. To counteract this situation, China is implementing a new type of high-quality urbanization aimed at urban development [67]. Therefore, we chose indicators to reflect the impact on the environment, as well as the quality of urbanization, which affects the speed and scale of urbanization and is especially important for the regions undergoing rapid development.

At present, the level of urban expansion is mainly represented by the evolution of construction land [68]. However, in terms of statistical data, urban built-up areas better reflect the scope of urban construction, so we used the urban built-up area to measure the level of urban expansion. The impact of urban land expansion on CO_2_ emissions has been analyzed in several studies [69,70,71]. It was found that urban expansion directly leads to the expansion of built-up areas and the loss of carbon storage; energy consumption also accelerates, and even the growth of carbon emissions is faster than urban expansion. Therefore, it is important to examine the effect of such factors on carbon emissions. However, population density and land-use level are less studied in terms of environmental impact or greenhouse gas emissions, which are the affecting factors focused on here. In the process of urbanization, not only changes in land use but also improvements in the level of use affect the regional environment [72]. We used the ratio of urban construction land to urban population to measure the level of land use. In addition, the degree of regional population agglomeration affects economic development as well as the ecological environment. In this study, population density was measured by the ratio of total resident population to built-up area.

#### 2.3.2. Decomposition of Factors

In this study, we used the LMDI based on the IPAT model to decompose the factors that affect carbon emissions for further analysis. This method has been widely used in carbon emissions decomposition analysis from the national level to the single-city level. Under the LMDI approach, the total change of CO_2_ emissions from time 0 to T can be decomposed as

(4) $$\Delta_{\mathrm{tot}}{\;=\;C}^{t}{\;-\;C}^{0}\;=\;\Delta A\;+\;\Delta G\;+\;\Delta \mathrm{In}\;+\;\Delta \mathrm{Ps}\;+\;\Delta S\;+\;\Delta \mathrm{De}\;+\;\Delta \mathrm{EI}\;+\;\Delta \mathrm{CI}$$

where ΔA is the urban expend effect, ΔG is the economic effect, ΔIn is the industrial structure effect, $\Delta P_{s}$ is the population structure effect, ΔS is the land use effect, ΔDe is the population density effect, ΔEI is the energy intensity effect, and ΔCI is the CO_2_ emissions intensity effect. The equations for these variables are as follows:

(5) $$\Delta A=\sum \frac{C^{t}{-C}^{0}}{{\mathrm{lnC}}^{t}{-\mathrm{lnC}}^{0}}\ln\left(\frac{A^{t}}{A^{0}}\right)$$

(6) $$\Delta G=\sum \frac{C^{t}{-C}^{0}}{{\mathrm{lnC}}^{t}{-\mathrm{lnC}}^{0}}\ln\left(\frac{G^{t}}{G^{0}}\right)$$

(7) $$\Delta \mathrm{In}=\sum \frac{C^{t}{-C}^{0}}{{\mathrm{lnC}}^{t}{-\mathrm{lnC}}^{0}}\ln\left(\frac{{\mathrm{In}}^{t}}{{\mathrm{In}}^{0}}\right)$$

(8) $$\Delta \mathrm{Ps}=\sum \frac{C^{t}{-C}^{0}}{{\mathrm{lnC}}^{t}{-\mathrm{lnC}}^{0}}\ln\left(\frac{{\mathrm{Ps}}^{t}}{{\mathrm{Ps}}^{0}}\right)$$

(9) $$\Delta S=\sum \frac{C^{t}{-C}^{0}}{{\mathrm{lnC}}^{t}{-\mathrm{lnC}}^{0}}\ln\left(\frac{S^{t}}{S^{0}}\right)$$

(10) $$\Delta \mathrm{De}=\sum \frac{C^{t}{-C}^{0}}{{\mathrm{lnC}}^{t}{-\mathrm{lnC}}^{0}}\ln\left(\frac{{\mathrm{De}}^{t}}{{\mathrm{De}}^{0}}\right)$$

(11) $$\Delta \mathrm{EI}=\sum \frac{C^{t}{-C}^{0}}{{\mathrm{lnC}}^{t}{-\mathrm{lnC}}^{0}}\ln\left(\frac{{\mathrm{EI}}^{t}}{{\mathrm{EI}}^{0}}\right)$$

(12) $$\Delta \mathrm{CI}=\sum \frac{C^{t}{-C}^{0}}{{\mathrm{lnC}}^{t}{-\mathrm{lnC}}^{0}}\ln\left(\frac{{\mathrm{CI}}^{t}}{{\mathrm{CI}}^{0}}\right)$$

## 3. Results

### 3.1. Characteristics of Land Urbanization

**Figure 2:**
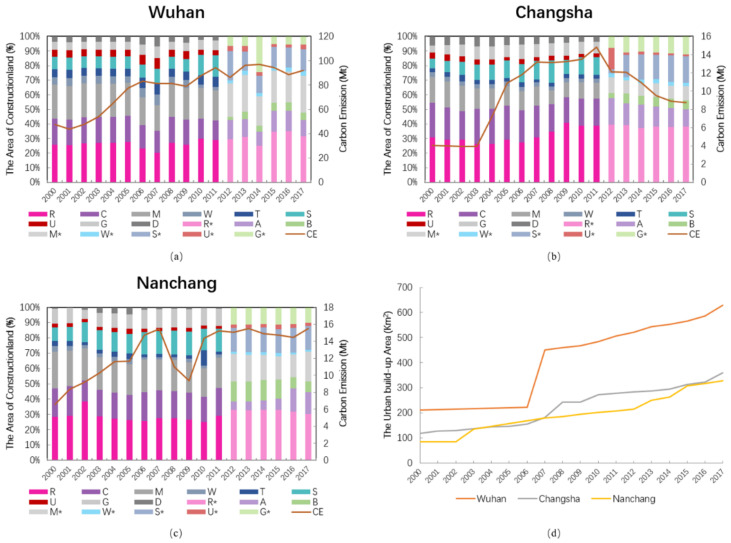
Area of construction land (The legend of construction land is as follows: 2000–2011: R–residential land, C–land for public facilities, M–industrial land, W–land for storage, T–land for transportation system, S–land for roads and plazas, U–land for municipal utilities, G–green land, D–land for special purposes. 2012–2017: R *–residential, A–administration and public services, B–commercial and business facilities, M *–industrial, manufacturing, W *–logistics and warehouse, S *–road, street, and transportation, U *–municipal utilities, G *–green space and square) and built-up in Wuhan, Changsha, and Nanchang. (**a**) Wuhan, (**b**) Changsha, (**c**) Nanchang, (**d**) Built-up area in Wuhan, Changsha and Nanchang.

Identifying the changes of the internal structure of construction land is of value to understanding land urbanization. As shown in Figure 2a–c, the overall changes in construction land area in Wuhan, Changsha, and Nanchang increased from 241.48, 118.82, and 83.85 km^2^ in 2000 to 840.19, 338.35, and 313.55 km^2^ in 2017, respectively. Construction land increased with annual growth rates of 11.17%, 6.64%, and 8.91%, respectively, which shows Wuhan is more prominent than Changsha and Nanchang. As opposed to the relatively flat growth in 2000–2005, Wuhan rose rapidly after 2005, followed by fluctuating growth. Note that Wuhan’s area decreased significantly after 2014 but recovered after 2016, whereas Changsha and Nanchang have grown steadily. From the perspective of land structure, residential land and industrial land are important components of construction land, accounting for 59.8%, 47.57%, and 49.18% of the total construction land in Wuhan, Changsha, and Nanchang during 2000 to 2017, respectively. Among them, residential land accounted for the largest proportion in all land types, increasing from 25.75%, 30.95%, and 28.35% in 2000 to 31.36%, 38.04%, and 31.71% in 2017, with an annual growth rate of more than 8%. Population migration was especially brought about by the process of urbanization, which caused the demand in land use to be higher than before. However, the industrial land shows clear regional differences. Obviously shown in Figure 2, between 2000 and 2017, only Wuhan’s industrial land area grew monotonically, whereas Changsha and Nanchang underwent fluctuating growth. Industrial land area increased by 156.73, 12.52, and 40.94 ha, respectively, for these cities. While the proportion of industrial land to the total showed different trends, Wuhan’s proportion increased from 23.27% to 25.34%. Conversely, Changsha and Nanchang showed decreases from 17.77% and 24.04% to 9.77% and 19.45%, respectively. Specifically, the area of industrial land increased whereas the proportion of construction land decreased, indicating that the internal structure is constantly adjusting.

According to the data statistics, built-up areas better reflect the status of urban expansion than does urban construction land. According to Figure 2d, the built-up area generally increased. However, the total carbon emissions underwent fluctuating growth in the early stage and then decreased starting in 2014. From 2000 to 2017, the built-up area of Wuhan, Changsha, and Nanchang increased by 24.59, 14.10, and 14.31 km^2^, respectively. Although Wuhan had the largest growth area, the average annual growth rate of Nanchang was 8.98% greater than that of the other two regions. The change in built-up area shows that the stages of urban expansion of the three regions differ slightly. Wuhan and Changsha expanded rapidly from 2006 to 2008, whereas Nanchang’s urban expansion uptick started in 2012. Compared with the stable growth stage, each region maintained a certain expansion speed after entering the rapid expansion stage.

### 3.2. Analysis of Carbon Emission and Influencing Factors

**Figure 3:**
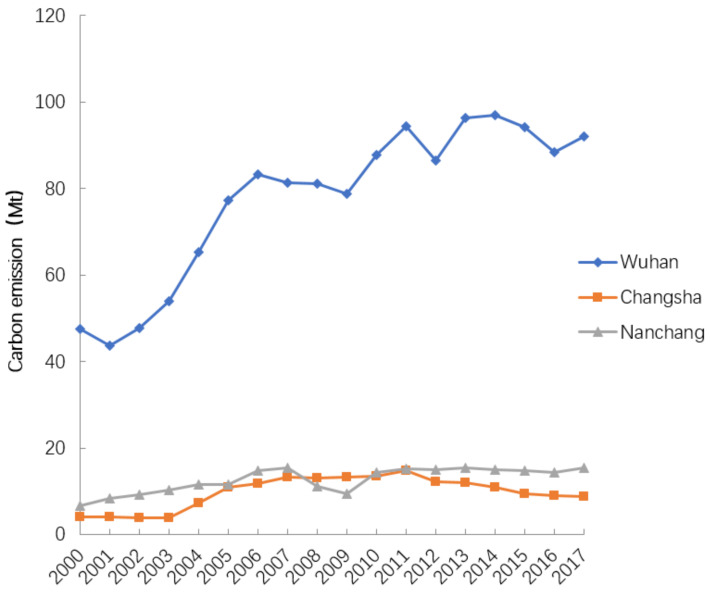
Total carbon emissions from Wuhan, Changsha, and Nanchang.

Figure 3 shows the change in CO_2_ emissions per city from 2000 to 2017. Due to the strong development of China’s economy and the increased energy consumption by industries located on construction land, the CO_2_ emissions in all regions gradually increased in varying degrees. During this period, the carbon emissions of Wuhan, Changsha, and Nanchang increased by 1396.85, 176.05, and 227.83 Mt, respectively. Wuhan’s CO_2_ emissions were significantly greater than those of the other regions; its annual average emissions were about seven times that of Changsha.

Table 2 shows the results for the elementary change in the LMDI analysis. Between 2000 and 2017, the greatest increase of built-up areas and urban construction land was recorded in Nanchang, while other areas lagged behind. The growth of urban construction land in Wuhan exceeded that in the built-up area, which has great construction potential. Over the past 18 years, the total population has also increased with a huge influx of population in the capital city itself, but the urban population proportion of Wuhan is only 4.52%, with little change over time when compared with 74.01% and 50.06% in the other regions. The GDP increased in all regions, with the greatest increase occurring in Changsha. Similarly, energy consumption also increased in all regions, with Wuhan posting the smallest change. The energy consumption intensity is trending downward in all cities, which is consistent with an elasticity coefficient of energy consumption less than 1.

### 3.3. Decomposition of Carbon Emission Factors

**Figure 4:**
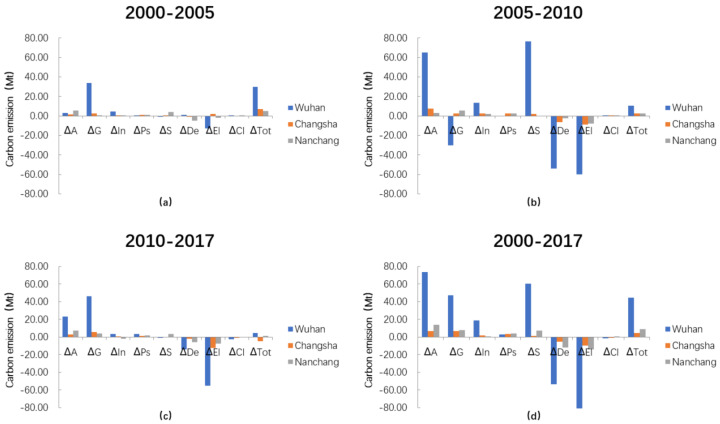
Factors influencing carbon emissions for (**a**) 2000–2005, (**b**) 2005–2010, (**c**) 2010–2017, and (**d**) 2000–2017 in the three regions.

From the perspective of direction of contribution to carbon emissions (i.e., promoting or curbing emissions), the temporal analysis of the regional characteristics shows that urban expansion, economy, industrial structure, population structure, and land use are the main forces driving the increase of carbon emissions. The change of population density effect, energy intensity effect, and carbon emissions effect helped to reduce the carbon emissions of the three cities between 2000 and 2017 by different degrees (Figure 4d). However, the carbon emission effect shows clear regional differences despite having a weak impact on carbon emissions themselves; its promotion of carbon emissions in Nanchang is in contrast to the other regions. We compared the results of different periods and divided the carbon dioxide emission characteristics of each city into three periods: 2000–2005, 2005–2010, and 2010–2017. As shown in Figure 4a–c, from 2000 to 2005, the energy intensity effect of Changsha; the land use effect, population density effect, and carbon emission effect of Wuhan; and the carbon emission effect of Nanchang showed opposing contributions to carbon emissions from the other periods. During 2005–2010, Wuhan’s economic effect, population structure effect, and carbon emission effect; Changsha’s carbon emission effect; and Nanchang’s land use effect and carbon emission effect were different from other periods in direction of influence. During 2010–2017, Wuhan and Changsha’s land use effect and Nanchang’s industrial structure effect were in the direction of curbing carbon emissions. We conclude that the main factors affecting carbon dioxide emissions followed similar directions, but some factors differed in their effects over the three periods.

In the context of urbanization, a strong correlation exists between China’s urban expansion, industrial production, energy consumption, and carbon emissions. Based on results, we are thus able to specify the impact magnitude of the different direction of factors on CO_2_ emissions. Urban expansion, economy, and energy intensity are the main factors driving changes in carbon emissions. Due to their national policies and respective economic situations, the regions have differences in economic output, energy consumption, and urban construction. This leads to differences in the magnitude of impacts on carbon emissions by the factors in the three periods; it is noteworthy that the fluctuation between 2005 and 2010 is larger than for the other two periods considered. During the period of 2000 to 2005, urban expansion, economic level, and energy intensity had an effect on carbon emissions of around 24.09, 5.98, and 4.27 Mt among the total change of carbon emissions of 29.79, 6.85, and 5.08 Mt, respectively, in Wuhan, Changsha, and Nanchang. However, from 2005 to 2010, the level of contribution decreased to −24.9, 0.99, and 0.69 Mt among the totals of 10.43, 2.62, and 2.66 Mt. During this period, China’s industrialization was accelerating, with the focus on the development of industries with high energy demand. At the same time, urban expansion caused by land urbanization affected the regional economic and industrial development. Compared with the previous period, the contribution level of three factors on carbon emissions rebounded to 14.66, −3.76, 3.75 Mt among the totals of 4.38, −4.76, and 1.18 Mt in the period of 2010 to 2017. Except for Changsha, the contribution level had a negative growth resulting from the overall reduction in carbon emissions. In general, the energy intensity effects in changing carbon dioxide emissions did not offset the effects of economic and urban expansion between 2000 and 2017. However, as time goes by, China continues to be transformed through industrial upgrading and new urbanization.

**Figure 5:**
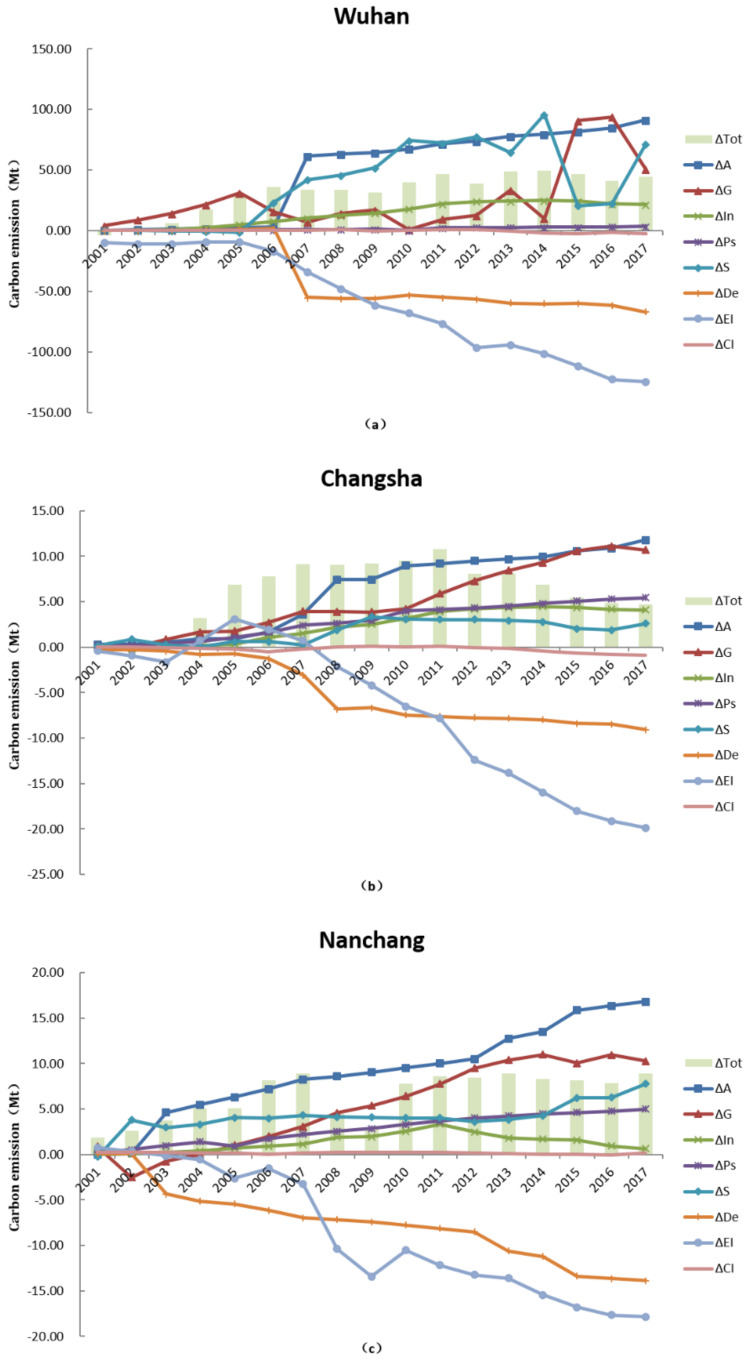
The factors causing carbon emissions in (**a**) Wuhan, (**b**) Changsha and (**c**) Nanchang by a time series.

The results of the LMDI analysis are also helpful in determining the main contributors in different stages and in showing the evolution of the degree of influence of various factors over different periods, as shown in Table 3. With the passage of time, the energy intensity effect of each region improved significantly. The overall trend of the temporal evolution is similar to that of the period analysis, but the characteristics vary by years and regions (Figure 5).

First, in Wuhan, the economic effect is the main reason for the increase in carbon emissions, with energy intensity offsetting only part of the increase. This indicates that the rapid growth of economic activity in a developing urban area leads to more carbon emissions. The GDP has exceeded 100 billion yuan in Wuhan since 2000, with a relatively large economy and a complete industrial system. It should be noted that this rapid economic growth was supported by tremendous energy consumption. However, as Table 3 shows, the economic effect contribution rate was negative in 2005–2010, and thus the result must be viewed cautiously. Comparing the change in carbon emissions with the contributions of the economic effect, it is found that the total CO_2_ emissions declined from 2006 to 2009 while the economic effect inhibited the emission reduction. In fact, this indicates that the level of economic development promoted the increase of carbon emissions in these years. On the other hand, the carbon emission effect had a positive contribution rate from 2000 to 2010, although it contributed little to reducing carbon emissions, probably due to the unreasonable utilization of energy structure, which led to more high-polluting energy consumption which aggravated the growth of carbon emissions faster than energy consumption at this stage. The population structure effect had a negative contribution caused by the volatile growth of urban population, leading to that factor increasing in contribution first and then decreasing, and which was relatively small compared to other factors in 2005–2010. The urban expansion effect has been rising steadily. However, since 2005, the rapid development and expansion of the city meant that construction land area increased rapidly, resulting in a sharp increase in urban expansion effect in 2006–2007. More recently, the contribution has increased steadily and exceeds the effect of the industrial structure. Land use and population density effects are worthy of further discussion: from 2000 to 2005, the land use effect contributed to decreasing carbon emissions, meaning that the reduction was caused by the improvement of land use level. Then, due to the change of urban land use in 2006, land use became a significant factor in the increase of carbon emissions. After 2006, the population density effect also contributed to decreasing carbon emissions with the rapid urban expansion and the decline in population density.

In Changsha, economic and urban expansion effects led to the increase of carbon dioxide emissions. From 2000 to 2017, the changes in GDP and energy consumption reached 759% and 141%, respectively (Table 2), meaning that high energy consumption was driving regional economic development while also promoting carbon emissions. The urban expansion effect contribution in 2005–2010 exceeded the economic effect due to the increase in indirect carbon emissions caused by the rapid urban expansion, with the continuous growth of construction land. Not only the energy intensity effect, but also the land use and population density effect offset the increase of carbon emission in 2010–2017. As shown in Figure 5, the cumulative contribution rate of the 2010–2016 land use effect continued to decrease. The growth rate of urban construction land was slower than that of urban population in this case. Compared with population density and energy consumption intensity, although the contribution of the land use effect was obviously weak, the negative contribution implies that the improvement in land use level has the effect of restraining carbon emissions. The energy intensity effect showed a positive contribution during 2000–2005. Figure 5 shows that 2003–2005 was the period with the fastest growth in energy consumption, and the continuous growth of energy consumption intensity in Changsha had a positive effect on carbon emissions. The carbon emission effect had a positive contribution in 2005–2010, meaning that the unreasonable utilization of energy structure led to an increase in energy consumption with high pollution and high emissions, so that the growth of energy consumption was less than the growth rate of carbon emissions. The effect of the population structure of Changsha is akin to urban expansion, and only lags behind urban expansion in the period analysis, especially from 2005 to 2010. Given the regional economic development, population structure has become an important force driving the increase in carbon emissions.

Urban expansion was also the main driving factor in carbon emissions in the Nanchang area; especially after 2010, it became more significant than the economic effect. Nanchang is considered to be the least developed region among those analyzed and is currently experiencing the stage of rapid industrialization and urbanization. Such a situation implies that the indirect carbon emissions acceleration caused by urban expansion has led to the development of industries in and around the region. The population density effect and energy intensity effect played a role in reducing carbon emissions. Although the changes are not significant, they reached their highest negative contribution in 2002–2003 and 2007–2008, respectively (Figure 5). Furthermore, the population density effect counteracted increased urban expansion effect in 2002–2003. Since 2011, the industrial structure effect has made a negative contribution to carbon emission and has increased over time. At the same time, the proportion of the industrial structure has also declined, indicating that the adjustment of industrial structure and reducing the proportion of industry have had a significant inhibitory effect on carbon emissions. Modifying the industrial structure should thus provide a good opportunity to reduce CO_2_ emissions in the future. The contribution of the carbon emission effect had a significant difference in Nanchang compared with the other regions, namely a positive contribution from 2000 to 2010. Moreover, the negative contribution in the latter period failed to offset the growth in carbon emissions (Figure 5). This reveals a tendency for high pollution energy consumption, which makes carbon emissions grow faster than energy consumption.

## 4. Discussion

From 2000 to 2017, the total construction land area and carbon emissions of the three main cities in the MYR-UA increased overall, with the growth rate of carbon emissions slowing or decreasing toward the end of this period compared to earlier in the period. This shows that carbon emissions do not always increase with the growth of construction land. From 2000 to 2010, the policies of urban agglomeration and economic and technological development zones accelerated regional urbanization. With Wuhan, Changsha, and Nanchang provincial capital cities as the core of growth, the development of the surrounding cities has been promoted, forming close spatial and economic urban agglomeration. Given the growing demands of population migration, infrastructure construction, and economic development, the increase in construction land leading to urban expansion was pronounced over the entire study period in Wuhan, Changsha, and Nanchang. Meanwhile, industries based on fossil fuel energy consumption were strengthened in order to achieve a higher level of industrialization, which led to the occupation of carbon sink land and consumption of a large amount of energy. Therefore, urban land use was extensive, which indirectly promotes carbon emissions. After 2014, the government issued the national new urbanization plan and the development plan for the MYR-UA [52,73]. The plan emphasizes the intensive and economical use of land and the smart growth of cities, with the large-scale use of construction land aiming to improve utilization efficiency and gradually reducing the industrial land to adjust the land structure. Further, the plan aims to improve the low-quality economic development which increases industrial output value based on expanding construction land. Consistent with the results in this study, the proportion of industrial land in Changsha and Nanchang has decreased gradually. On the other hand, although the proportion of industrial land in Wuhan increased due to the large volume of the economy, the magnitude of increase is small. In addition, industrial adjustment and production technology improvement would also have an impact. The government has made great efforts to develop the tertiary industry in order to reduce the proportion of industry. Measures have also been pursued such as developing low-carbon technology, promoting energy saving technology, and popularizing low-carbon products to improve energy efficiency in the aim to achieve reduction. These indirect effects have eased the growth of carbon emissions. Therefore, the growth of the two curves differ, consistent with the results of related research [66], which pointed out that an inverted U-shaped relationship exists between the total construction land and carbon emissions. As the built-up area expands, the carbon emission rate gradually decreases.

The following findings were obtained through the decomposition analysis results:

(1) As reported in the decomposition literature [74,75], energy intensity is the main reason for the reduction in total carbon emissions, which means that improved energy intensity plays an important role in reducing carbon emissions. Over the period, energy intensity has become an important constraint index of national economic development. This indicates that local governments should pay attention to ecological environment protection while strengthening urban construction. Meanwhile, the guiding programs related to energy conservation and emission reduction have been implemented, such as *Comprehensive Work Plan for Conserving Energy and Reducing Emissions*. Energy-saving advanced technology has been widely used. In addition, at the regional level, Nanchang, Wuhan, and Changsha have been listed as low-carbon pilot cities to promote low carbon levels in production and consumption since 2010. The implementation of these policies has played a positive role in improving energy efficiency and reducing carbon emissions. This is consistent with the reduction of energy intensity in our results.
(2) So far, conflicting opinions exist on the question of the influence of population density, with some research concluding that population density in urban areas is a positive factor in reducing carbon emissions [8,60,76]. These studies pointed out that increases in population density combined with compact urban development will generate scale effects, which contribute to improving energy savings as well as the efficiency of energy intensive facilities, eventually cutting down carbon emissions. On the contrary, some research has concluded that the decline of population density has an inhibitory effect on carbon emissions [31,32,77], which is similar to our research results. This is likely due to the idea that urban areas have not yet formed a compact development model, and the advantages of scale effect have not been reflected during the period, indicating that there is still much room for improvement in local planning and management.
(3) Economic level is considered the main factor in the increase of carbon emissions [78]; that is generally consistent with our results. The Chinese accession to the World Trade Organization (WTO) in 2001 stimulated the economic development of capital cities such as Wuhan, Nanchang, and Changsha. At the same time, the population transfer to urban areas and the progress of industrialization also increased energy consumption that in turn promoted CO_2_ emissions. One explanation for this is that local governments have pursued rapid GDP growth by simply expanding the scale of production, forming an extensive growth mode sequentially. This development model promotes economic growth in the short term while also promoting pollution by high-energy-consuming industries [79]. However, under the general trend of a significant slowdown in economic growth rate after entering into a new-normal economy, the development mode relying on energy intensive industries should be gradually replaced by green industry. The reduction of energy consumption will thereby reduce the total carbon emissions.
(4) As with economic level effect, the results of the time-series and period analysis in this study show that the urban expansion effect makes a significant contribution to carbon emissions. This differs from the results of other decomposition analysis studies [25,31]. A possible reason for this discrepancy is that the economic effect in this study is expressed in terms of GDP per unit area, whereas the growth of construction land exceeds that of GDP, which allows the urban expansion effect to promote carbon emissions more than the economic effect. Moreover, it also shows the prominent positive impact of urban expansion on raising regional carbon emissions. Land urbanization in the capital city has tended to be accompanied by a large-scale population migration to the urban area that increases urban population. This migration has improved large-scale public infrastructure construction [8], which in turn stimulated demand for fossil-fuel-based energy. In addition, the industrial production scale required in order to achieve the sustained economic growth witnessed in these cities has consumed land resources and increased carbon emission levels. Finally, the resulting rapid urban expansion has led to changes in land use structure and subsequent decreases in the amount of land able to serve as a carbon sink. Therefore, the finding of increases in carbon emissions from urban expansion is consistent with our expectations.
(5) Compared with the significant trend in economic level and urban expansion, the industrial structure effect exhibited weaker trends. Rapid industrialization is a driver of fossil-fuel energy use, a development that leads to rapid increases in carbon emission. Especially after the implementation of *The Rise of Central China*, the urban industrial GDP growth rate has increased with the proportion of industry growth. In contrast, the decline of the industrial proportion by the adjustment in industrial structure was found to play a significant role in decreasing CO_2_ emissions.
(6) The results of this study show that population structure and land use have different effects on regional carbon emissions in different stages. An increase in total population generally has a positive impact on carbon emissions while inhibiting carbon emissions with the increases in the proportion of population structure [8]. It is perhaps unsurprising that rapid economic growth in capital cities has stimulated increase in the income of urban population, concretizing a tendency in clean energy consumption of urban people with the transition from traditional fossil fuels to clean energy sources. Given this, carbon emissions will decrease by the higher energy efficiency of clean energy sources. However, the results of this study indicate that when the urban population growth is less than that of the total population, the population structure effect makes a negative contribution (i.e., decreases carbon emissions). This demonstrates that the current energy consumption habits of urban population have not shifted to clean energy consumption, and the increase of population structure rate will not offset the increase of carbon emissions. Regarding the increase in construction land, it is closely related to economic development and population scale [80]. Given stable population growth, the rapid growth of construction land will lead to a significant positive contribution from land use, which means extensive use of land resources leading to an increase in carbon emissions. On the other hand, carbon emissions growth can be mitigated by improving land-use efficiency.

Finally, this study could be improved in the following ways: (1) spatio-temporal land use data could be used as the data source for urban land, allowing for an accounting of regional carbon emissions based on land use. We could then more precisely analyze how construction land affects carbon emissions and put forward suggestions on adjusting the land use structure accordingly to mitigate carbon emissions. (2) Energy consumption data for calculating carbon emissions from the traditional energy statistics are primarily concentrated on the national level, provincial level, or the level of mega cities. In the published statistics, city-level data of energy consumptions such as the balance sheet of energy consumption are usually vacant for the general cities, leading to most of the energy-related carbon emissions at the city level coming from industrial consumption. Therefore, future work should consider the energy consumption data from human activities in construction land to more accurately estimate the carbon emission from different sectors. (3) This study analyzed the magnitude of impact of various factors on carbon emissions only descriptively through the LMDI decomposition model. The limitation of LMDI motivates future research to investigate the causal relationship of carbon emission and influencing factors through econometrics. (4) The target for carbon emission reduction in 2020 has been met, but the latest data should be updated for phased comparative analysis and research before the peak carbon emission target is reached by 2030.

## 5. Implications of Results

The results show that energy intensity plays a key role in reducing carbon emissions. To reduce the per unit GDP of energy consumption intensity, the government should first focus on the development of clean energy technology and improve the structure of energy consumption. Energy efficiency should be improved by upgrading energy technology and reducing the dependence of industry on high energy consumption. Eliminating industries that lead to high energy consumption is another way to reduce emissions. By optimizing the industrial structure and promoting the development of a low-carbon economy, the sustainable development of energy, economy, and the environment can be coordinated. Given that Wuhan, Changsha, and Nanchang are central urban agglomerations in the middle reaches of the Yangtze River, the implementation of corresponding policy should help their surrounding cities to transition to a low-carbon economy.

Furthermore, controlling the growth of city scale and reducing the expansion of construction land is an effective means to reduce carbon emissions. To some extent, unnecessary production and construction activities can be avoided by limiting the conversion to construction land from other types of land with a carbon-sink capacity. The impact on carbon emissions of expanding construction land differs among regions. The expansion of land use should be controlled to the maximum extent according to the actual situation. This strategy should not inhibit economic development but effectively reduce carbon emissions and ensure the sustainable development of the region.

## 6. Conclusions

This study analyzed the land urbanization and carbon emissions of three central cities in the middle reaches of the Yangtze River urban agglomeration. The results show that the built-up area grew monotonically from 2000 to 2017 and that the study area is in the rapid development stage of land urbanization. The total carbon emissions from the study area are increasing, with Wuhan accounting for the largest increase in carbon emissions (44.60 Mt). The carbon emissions of Changsha began to decline, whereas those of Nanchang remained stable, from 2011.

In addition, we analyzed quantitatively how various factors affect regional carbon emissions over a period and temporal time. The period analysis revealed three stages: 2000–2005, 2005–2010, and 2010–2017. In all stages, energy intensity had the most significant inhibitory effect on carbon emissions. Contrarily, the population density effect of Changsha and Nanchang in 2000–2005 produced the greatest reduction in carbon emissions. The economic effect is the key factor that increases regional carbon emissions, and the economic effect of Wuhan and Changsha was more apparent than that of Nanchang. In addition, for Wuhan and Changsha, urban expansion increased carbon emissions to a degree second only to the economic effect, whereas it claimed the top position for Nanchang. Moreover, the results for 2000–2017 show that urban expansion in Wuhan and Nanchang increased carbon emissions to a greater extent than did the economic effect. The temporal analysis shows that, although the annual contributions to carbon emissions vary as the factors themselves change over time, the cumulative contribution to carbon emissions remains basically consistent with the period analysis.

Based on these results, we argue that energy intensity is the main factor contributing to reductions in carbon emissions, followed by population density. The economic effect and urban expansion effect contribute the most to increasing carbon emissions. Finally, the effect on carbon emissions from industrial structure, population structure, and land use vary over the different stages and regions of this study.

## Figures and Tables

**Table 1:** Definition of variables used in this study.

Variable		Definition	Unit of Measurement
CO_2_	CO_2_ emissions	Total carbon dioxide emissions from energy consumption of industrial enterprises above Designated size	Million tons (Mt)
A	Urban Expansion	Area of urban build-up	Square kilometer (km^2^)
G	Economic level	GDP divided by urban construction land	Ten thousand yuan/Square kilometer
In	Industrialization level	Share of industrial value-added in GDP	Percent
Ps	Population structure	Proportion of people living in urban areas which is end-year resident population divided by total population	Percent
S	Land utilization level	Urban construction land per resident population	Square kilometer/number of people
De	Population density	end-year resident population per area of urban built-up	number of people/Square kilometer
EI	Energy intensity	Energy use per unit industrial value-added GDP	Ten thousand tons standard coal/Ten thousand yuan
CI	CO_2_ emissions intensity	CO_2_ emissions per unit energy use	Ten thousand tons/Ten thousand tons standard coal

**Table 2:** Elementary change in three regions for 2000–2017 (%).

	Urban Land		Population		GDP		Energy Consumption	Energy Intensity	CO_2_ Emissions	Elasticity
	B-land	C-land	Total	Structure	Total	Per Area				
Wuhan	199.10	247.93	35.35	4.52	603.46	102.18	98.27	−78.67	93.80	0.16
Changsha	201.72	184.76	28.99	74.01	759.25	201.75	140.71	−79.91	116.7	0.19
Nanchang	289.7	273.94	26.13	50.06	694.49	112.46	129.29	−73.49	135.84	0.19

Elasticity is measured using the change of energy consumption and total gross domestic production (GDP) in Table 2, where elasticity = EC/GDP_tot._

**Table 3:** Contribution of different factors to carbon emission from the three regions (units are %, Mt).

**City**	**Stage**	$C_{A}$	$C_{G}$	$C_{In}$	$C_{Ps}$	$C_{S}$	$C_{De}$	$C_{EI}$	$C_{CI}$	ΔTot
Wuhan	2000–2005	9.77	114.18	16.01	13.86	−15.48	3.39	−43.08	1.35	29.79
	2005–2010	622.61	−287.79	127.99	−46.10	774.83	−518.67	−573.65	0.78	10.43
	2010–2017	535.66	1053.35	80.27	77.75	−15.39	−315.28	−1253.98	−62.39	4.38
Changsha	2000–2005	20.77	37.91	5.13	19.06	8.67	−16.67	28.65	−3.51	6.85
	2005–2010	289.27	93.78	104.26	105.92	87.72	−244.50	−345.40	8.95	2.62
	2010–2017	−63.26	−121.17	−13.05	−31.44	8.55	36.21	263.54	20.62	−4.76
Nanchang	2000–2005	109.14	11.95	11.14	18.40	76.47	−94.56	−37.08	4.54	5.08
	2005–2010	121.23	207.95	71.50	91.70	−4.71	−86.99	−303.35	2.66	2.66
	2010–2017	613.37	324.86	−159.19	141.18	319.09	−514.72	−620.36	−4.23	1.18
